# The Antarctic Moss *Pohlia nutans* Genome Provides Insights Into the Evolution of Bryophytes and the Adaptation to Extreme Terrestrial Habitats

**DOI:** 10.3389/fpls.2022.920138

**Published:** 2022-06-17

**Authors:** Shenghao Liu, Shuo Fang, Bailin Cong, Tingting Li, Dan Yi, Zhaohui Zhang, Linlin Zhao, Pengying Zhang

**Affiliations:** ^1^Key Laboratory of Marine Eco-Environmental Science and Technology, First Institute of Oceanography, Ministry of Natural Resources, Qingdao, China; ^2^School of Advanced Manufacturing, Fuzhou University, Jinjiang, China; ^3^National Glycoengineering Research Center, School of Life Sciences and Shandong University, Qingdao, China

**Keywords:** whole-genome sequencing, gene-family expansions, Antarctic bryophyte, metabolomic profiling, flavonoid biosynthesis, UV-B radiation

## Abstract

The Antarctic continent has extreme natural environment and fragile ecosystem. Mosses are one of the dominant floras in the Antarctic continent. However, their genomic features and adaptation processes to extreme environments remain poorly understood. Here, we assembled the high-quality genome sequence of the Antarctic moss (*Pohlia nutans*) with 698.20 Mb and 22 chromosomes. We found that the high proportion of repeat sequences and a recent whole-genome duplication (WGD) contribute to the large size genome of *P. nutans* when compared to other bryophytes. The genome of *P. nutans* harbors the signatures of massive segmental gene duplications and large expansions of gene families, likely facilitating neofunctionalization. Genomic characteristics that may support the Antarctic lifestyle of this moss comprise expanded gene families involved in phenylpropanoid biosynthesis, unsaturated fatty acid biosynthesis, and plant hormone signal transduction. Additional contributions include the significant expansion and upregulation of several genes encoding DNA photolyase, antioxidant enzymes, flavonoid biosynthesis enzymes, possibly reflecting diverse adaptive strategies. Notably, integrated multi-omic analyses elucidate flavonoid biosynthesis may function as the reactive oxygen species detoxification under UV-B radiation. Our studies provide insight into the unique features of the Antarctic moss genome and their molecular responses to extreme terrestrial environments.

## Introduction

The features of the Antarctic terrestrial environment are characterized by high ultraviolet radiation, freezing, and extreme dryness (Perera-Castro et al., 2020; Cassaro et al., 2021). Notably, the strong ultraviolet radiation in Antarctica is a typical consequence of global climate change and human activities. Enhanced UV-B radiation (280–315 nm) has been widely concerned since the 1980s due to ozone depletion, which results from the breakdown of chlorofluorocarbons (CFCs) in the atmospheric stratosphere (Hossaini et al., 2017; Neale et al., 2021). Due to the Montreal Protocol and its amendments prohibiting the release of harmful CFCs, the ozone depletion seems to have slowed down. However, ozone hole run up to 24.8 × 10^6^ km^2^ in 2021 and enlarged to most regions of the Antarctic continent. The Antarctic terrestrial plants are undergoing the higher UV-B light of 3.4–6.2 mW/cm^2^ (Bao et al., 2018). They may battle the UV-B radiation by producing antioxidants such as flavonoids and other UV-B-absorbing pigments, modulating reactive oxygen species (ROS)-scavenging enzyme activities (Pereira et al., 2009; Singh et al., 2011; Singh and Singh, 2014; Wang et al., 2020). The field experiments demonstrated that continuing UV-B radiation reduced the chlorophyll contents in the Antarctic moss (*Bryum argenteum*) and lichen (*Umbilicaria aprina*), and increased the contents of UV-B absorbing compounds (Singh and Singh, 2014). The Multi-omics analysis indicated that UVR8-mediated signaling, flavonoid biosynthesis, and DNA repair machinery might facilitate the adaptation of Antarctic moss to UV-B radiation (Li et al., 2019; Liu et al., 2021). The moderate moss *Physcomitrella patens* was more qualified of surviving UV-B radiation than Arabidopsis, and several flavonoid biosynthesis genes were also markedly upregulated in response to UV-B radiation (Wolf et al., 2010). However, compared with other land plants, the molecular mechanism of bryophytes acclimation to strong UV-B radiation was far less documented.

Antarctica is considered to be the coldest continent on Earth. The daily values of air temperature over 0°C are only achieved for the few short summer months or weeks (Convey et al., 2018). Field experiments showed that the moss surface temperatures were over +4°C for 43.4% of the peak Antarctic summer, while surface temperatures exceeded 14°C for an average of just 2.5% of the time (Perera-Castro et al., 2020). Antarctic mosses also underwent a higher frequency of air freeze-thaw cycles during the austral summer. However, the regional climate in maritime Antarctica such as the South Shetlands Islands is relatively milder, with temperature ranges from −5°C to 13°C in the summer daytime (Perera-Castro et al., 2020). Furthermore, Antarctic mosses usually have surface temperatures well above air temperature, over 15°C at midday in summer, relying on the water content of moss tundra. Antarctic mosses propagating in this cold habitat largely depend on the capacity to maximize photosynthesis for short summer and reduce respiratory carbon losses (Perera-Castro et al., 2020).

Most regions of the East Antarctica are increasing dryness due to global climate change. In recent decades, the more positive Southern Annular Mode, driven by ozone depletion and greenhouse gas emissions, maintains freezing temperature in East Antarctica (Robinson et al., 2018). Subsequently, the freezing temperature causes the drying climate in the East Antarctica. Vegetation distributions are markedly influenced by local availability of ice-free lands and water. For instance, in Windmill Islands, East Antarctica, moss communities are the well-developed and extensive; but the health of moss-beds is declining due to regional dryness (Malenovský et al., 2015). Drought stress usually causes photoinhibition and photodamage of photosynthetic apparatus with growth retardation and yield reduction.

Global climate change and human activities are having a significant impact on the Antarctic terrestrial ecosystem (Convey and Peck, 2019; Cannone et al., 2022). The Antarctic Peninsula is now experiencing some of the most rapid warming on earth (Sato et al., 2021). The ice-free areas of Antarctica is expected to increase by approximately 25% by the end of 21st century due to climate change, while most of this expansion will occur in the Antarctic Peninsula (Lee et al., 2017). Warming had caused the special phenomena of flowering plant spread and snow algae outbreak (Gray et al., 2020; Cannone et al., 2022). Similarly, a significant increase was found in biological activity over the past 50 years, measured through moss growth or accumulation rates (Amesbury et al., 2017). Currently, Antarctic plants appears to be promising models for studying the adaptation mechanism to various abiotic stresses (Convey and Peck, 2019; Perera-Castro et al., 2020; Bertini et al., 2021; Liu et al., 2021) and monitoring the regional climate changes (Amesbury et al., 2017; Lee et al., 2017; Robinson et al., 2018; Cannone et al., 2022) as well as assessing the impact of human activities (Malenovský et al., 2015; Hughes et al., 2020). Particularly, the genomic features were widely clarified in the Antarctic bacteria (Benaud et al., 2021), algae (Zhang et al., 2020b), and fishes (Kim et al., 2019), but the underpinnings remain unclear in the adaptation of mosses to extreme environments.

Mosses and lichens are the dominant floras in the coastal regions of Antarctica (Cannone et al., 2016; Bertini et al., 2021). The moss *Pohlia nutans* is abundant in the Fildes Peninsula and Victoria Land of Antarctica where water supply is plentiful (Skotnicki et al., 2002; Liu et al., 2019; Wang et al., 2020). They usually occur as small and isolated colonies with short shoots (1–2 cm length). Similar to other mosses in these regions, they appear to reproduce asexually through dispersal of vegetative propagules-small fragments of colonies (Skotnicki et al., 2002). Protonema proliferation plays an essential role in the asexual processes of differentiation and regeneration (Zhao et al., 2019). Since bryophytes commonly possess most key innovations of land plant evolution, the Antarctic psychrophilic mosses represent an emerging model system for studying responses and sensitivities to environmental changes (Convey and Peck, 2019). Here, the whole-genome sequencing, transcriptome and metabolome profiling, as well as comparative genomic analysis will enlarge our understanding of early land plant evolution and the adaptations of these basal land plants to the polar terrestrial environments.

## Materials and Methods

### Plant Materials and Stress Treatments

The moss *P. nutans* isolate NO.L were gathered from the Fildes Peninsula of Antarctica (S62°13.260′, W58°57.291′), in March 2014. They were cultivated under conditions according to previous reports (Wang et al., 2020; Liu et al., 2021). For cold stress, mosses were placed under 0°C. For mock drought stress, mosses were treated with 20% PEG6000. Two Philips TL20W/01RS narrowband UV-B tubes were used for UV-B light source as described previously (Wolf et al., 2010; Liu et al., 2021). Mosses without treatments or treated with sterile water were used as control group. The green parts of gametophytes were collected and used for genome and transcriptome sequencing, and UPLC-MS/MS analysis.

### Estimation of Genome Size

Genomic DNA was prepared from the gametophytes of *P. nutans* using a modified cetyltrimethylammonium bromide (CTAB) method (Allen et al., 2006). DNA quality was detected using a Nanodrop 2000 Spectrophotometer (Thermo Fisher Scientific, United States) and an Agilent BioAnalyzer (Agilent, United States). DNA samples were broken into fragments with a length of 350 bp by an ultrasonic disruptor. DNA library was constructed and subjected to Paired-end sequencing using the Illumina Hiseq sequencing platform. Finally, clean reads were used to estimate the *P. nutans* genome size using *K*-mer frequency (*K*-mer = 17 bp; Zhang et al., 2020b).

### Library Construction and Genome Sequencing

The paired-end genomic libraries were constructed using the Illumina TruSeq DNA PCR-Free Prep kit and sequenced with an Illumina HiSeq × 10 platform; the long inserts of SMRT Cell libraries were constructed and sequenced with a PacBio Sequel II instrument (Pacific Biosciences, CA, United States); the chromatin interaction mapping (Hi-C) libraries were constructed with 350–500 bp insertions and sequenced on an Illumina HiSeq platform × 10 platform. The quality of the Hi-C sequencing was assessed using the HiCUP pipeline (Wingett et al., 2015). Raw reads were trimmed to discard adaptor sequences, potential contaminants, and others.

### Genome Assembly and Quality Assessment

We utilized the PacBio SMRT-sequencing and Hi-C-based scaffolding approaches with further polishing using Illumina short reads to assemble a high-quality of the *P. nutans* genome. In brief, 31.16 Gb of PacBio single-molecule long reads (average length, 13.73 kb) from SMRT sequencing were assembled into contigs using hifiasm (Cheng et al., 2021). The Illumina clean short reads obtained previously were aligned to the PacBio assembly using BWA (Li and Durbin, 2009) and Minimap2 (Li, 2018). The repetitive polishing was conducted using Pilon (v.1.22; Walker et al., 2014). The assembled contigs were submitted to BLAST search against the NCBI non-redundant (NR) nucleotide database to discard organellar DNA and prokaryotic sequences. Then, we combined the assembled contigs from PacBio sequencing and Illumina clean short reads into scaffolds using SSPACE (v.3.0) tool (Boetzer et al., 2010). Finally, 63.26 Gb of Hi-C sequencing clean reads were employed to cluster, orientate, and link the assembled scaffolds into 22 pseudo-chromosomes using HiCUP pipeline (Wingett et al., 2015). The completeness of genome assembly was evaluated by alignment with the plantae database of Benchmarking Universal Single-Copy Orthologs (v.3; Manni et al., 2021). Gene region completeness was assessed using transcriptome data of the Antarctic moss *P. nutans*.

### Repeat Annotation

Repeat sequences annotation was carried out using a combined method of Repeatmasker, Proteinmask, and *de novo* that were described in previous publication (Zhang et al., 2020a). For the *de novo* approach, LTR_FINDER (Xu and Wang, 2007), PILER (Edgar and Myers, 2005), and RepeatModeler (Flynn et al., 2020) were used for TEs prediction and build the *de novo* repeat sequence libraries. Then, RepeatMasker was conducted to search for repeats in the *P. nutans* genome refer to the *de novo* repeat libraries as reference libraries. For the homology-based approach, TEs were identified using RepeatMasker and RepeatProteinMask with the integrate Repbase database of known repeat sequences and the *de novo* repeat sequence libraries (Zhang et al., 2020a). The final non-redundant repeat sequences were obtained by integrated together overlapping TEs from both *de novo* and homology-based predictions. The transposable elements (TEs) were calculated and summarized in the *P. nutans* genome.

### Insertion Time of LTR Retrotransposon

Full-length LTR-RTs were further investigated from the *P. nutans* genome assembly using LTRharvest (Ellinghaus et al., 2008) and LTRretriever (Ou and Jiang, 2018). Default parameters were used except for -minlenltr 100 -maxlenltr 7000 -mintsd 4 -maxtsd 6 -motif TGCA -seqids yes. *Copia* and *Gyspy* in all non-redundant LRT-RTs file were used for tree construction. Full-length LTRs were aligned with MAFFT (Katoh and Standley, 2013). The insertion time of LRT-RTs was calculated by LTRretriever.

### Whole-Genome Duplication Incidents

To find the potential whole-genome duplications (WGD), the *P. nutans* genome was self-aligned or aligned with *P. patens* to identify duplicated gene pairs. All *K*s (rate of synonymous substitutions) distributions were calculated using wgd command-line tool (v.3.0; Zwaenepoel and Van De Peer, 2019). All *K*s values ≤0.1 were removed to avoid the incorporation of allelic and/or splice variants (Lang et al., 2018). A rough dating procedure of the WGD event were conducted based on the observed sequence divergence using the *K*s of 0.11 and a referenced substitution rate (*r*) of 9.4 × 10^−9^ per synonymous site per year in *P. patens* (Rensing et al., 2008). The time gene insertion (*T*) was calculated using the formula *T* = *K*s/2*r*.

### Gene Prediction and Functional Annotation

Protein-coding genes were predicted from the *P. nutans* genome using three strategies: (1) *de novo* prediction, (2) homology-based prediction, and (3) RNA-sequencing annotation. For *de novo* prediction, AUGUSTUS (v.2.5.5) and GlimmerHMM (v3.0.1) were used to detect genes (Majoros et al., 2004; Stanke et al., 2006). For homology-based prediction, proteins of five well-annotated species (*P. patens*, *Amborella trichopoda*, *Ananas comosus*, *Abrus precatorius*) were aligned to the *P. nutans* genome using TBLASTN, with a threshold E-value ≤1 × 10^−5^. The resultant alignments were analyzed using Genewise (v.2.4.1; Birney et al., 2004). The *de novo* assembly and homologues results were incorporated in MAKER to generate a consensus dataset (Holt and Yandell, 2011). To supplement the dataset, we aligned the RNA-sequencing data to the genome using TopHat (v2.1.1), and the alignments were used as input for Cufflinks (v.2.2.1; Trapnell et al., 2009, 2012). We merged the MAKER consensus and transcripts to generate the final dataset. Gene functional annotation was conducted according to best hit of BLASTP (*E*-value ≤1E-05) against SwissProt, Non-Redundant, TrEMBL, and KEGG databases.

### Evolution Analysis

To build the dataset for gene-family clustering, we obtained the protein-coding genes from the genomes of 14 green plants, containing five bryophytes (the Antarctic moss *P. nutans*, the moss model plant *P. patens*, the liverwort model plant *Marchantia polymorpha*, the hornwort model plant *Anthoceros angustus*, and the moss *Ceratodon purpureus*). We only used the longest transcript of each gene. Orthogroups or gene families were detected after conducting an all-against-all BLASTP search using OrthoMCL (v.2.0, threshold *E*-value of 1.0 × 10^−5^; Li et al., 2003). A venn diagram was present by comparison of gene families identified from different plants. The syntenic blocks were identified using MCScanX or jcvi software (threshold *E*-value ≤1.0 × 10^−5^; Wang et al., 2012).

We constructed species phylogenetic tree using single-copy orthologs. Since the *P. nutans* genome underwent a recent WGD events with few single-copy orthologs, we therefore used half of the paired chromosomes of *P. nutans* for species evolution analyses. The single-copy orthologs were aligned using MAFFT and finally joined into the super alignment matrix (Katoh and Standley, 2013). Then, the maximum likelihood phylogenetic analyses were conducted by RAxML (v.7.2.3; Stamatakis, 2006). Divergence times were calculated using MCMCTree in PAML v4.9 (Yang, 2007). Calibration points on the tree branches were inferred from Timetree.^1^ The time-calibrated phylogeny was mainly congruent with previous phylogenetic analyses (Zhang et al., 2020a). Gene-family sizes were estimated using the OrthoMCL program. The gene family was filtered by threshold of variance/mean < 10 and missing rate < 25%. The gene family expansion or contraction were calculated using CAFÉ (v2.1) based on the maximum likelihood model (*q-*value ≤0.05; De Bie et al., 2006). The expanded gene families were analyzed by KEGG pathway enrichment.

### Transcription Factor Annotation

We utilized the plant transcription factors (TFs) prediction program iTAK (v.1.7) to identify TFs (Zheng et al., 2016).

### Identification and Phylogenetic Analysis of Gene Families

Hmmsearch program was used to identify the L-phenylalanine ammonia-lyase (PAL), chalcone synthase (CHS), 2-oxoglutarate-dependent dioxygenase (2-OGD), DNA photolyase, and other family proteins from five bryophyte genomes with default parameters of --cut_tc. Sequence alignments were conducted using MAFFT (Katoh and Standley, 2013). Phylogenetic trees were constructed with IQ-TREE software (v1.6.12) from amino-acids sequences (with maximum-likelihood method, 1,000 replicates, and LG + F + R7 model; Nguyen et al., 2015).

### Transcriptome Sequencing

Mosses were treated with UV-B light, Cold (0°C), and drought (20% PEG6000). RNA isolation and transcriptome sequencing were conducted following the procedure described previously (Li et al., 2021). The gene expression levels were estimated with RPKM (Reads Per Kilobase per Million mapped reads). The |log_2_(Treat/Control)| > 1 and FDR (*q-*value, Corrected *p*-value) < 0.05 were used as the threshold to discriminate the differentially expressed genes (DEGs).

### Metabolomics Profiling and Analyses

Mosses were treated with UV-B light for 3 days. The moss gametophytes from UV-B radiation groups (i.e., UV-B) and control groups (i.e., CG) were cut and used for metabolite analysis. For cold stress, mosses were placed under 0°C for 60 h. The metabolite extraction, qualitative, and quantitative analysis were performed in accordance with previously described methods (Li et al., 2021; Wang et al., 2021b).

### Statistical Data Analysis

Data were statistically compared between different groups and the statistical significance were calculated using Student’s *t* test (^*^p < 0.05, ^**^p < 0.01). For multivariate data analysis of metabolome, principal component analysis (PCA), hierarchical clustering analysis (HCA), and orthogonal projections to latent structure-discriminant analysis (OPLS-DA) were performed according to earlier publications (Nyamundanda et al., 2010; Li et al., 2021). Metabolites with log_2_ |fold change| ≥ 1 and VIP ≥ 1 were considered as differentially changed metabolites between two groups.

## Results

### Genome Assembly and Annotation

**Figure 1:**
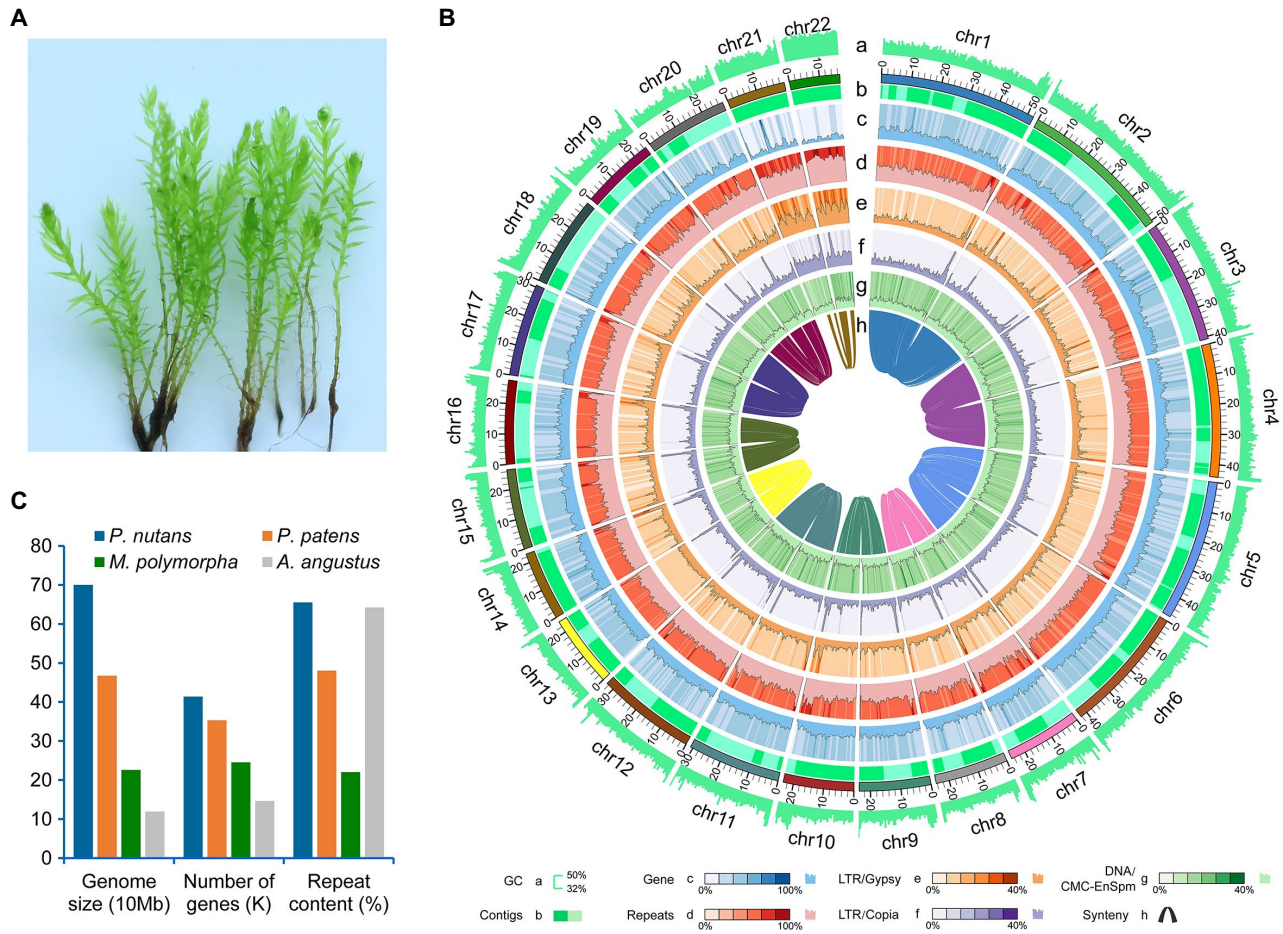
Genomic structure of the Antarctic moss *Pohlia nutans*. **(A)** Photo of *P. nutans* under normal condition. **(B)** The landscape of genome assembly and annotation of *P. nutans*. Tracks from outside to inside correspond to: a, GC content; b, contigs density; c, gene density; d, repeat density; e, LTR/*Gypsy* content; f, LTR/*Copia* content; g, DNA/CMC-EnSpm; and h, syntonic relationship. **(C)** The genome size, gene content, and repeat content of four bryophyte genomes (the Antarctic moss *P. nutans*, the moss model plant *Physcomitrella patens*, the liverwort model plant *Marchantia polymorpha*, the hornwort model plant *Anthoceros angustus*).

The moss gametophytes were used for genome and transcriptome sequencing (Figure 1A). To obtain a high-quality genome of *P. nutans*, we used a combination of Illumina short-read and PacBio Sequel II HiFi-read sequencing approaches. The estimated genome size was 708 Mb, and the proportion of repeat sequences 62.02%, based on the distributions of *K*-mer frequency. We employed a hierarchical assembly approach using 31.16 Gb (44.01-fold coverage) of PacBio HiFi reads, 83.00 Gb (131.36-fold coverage) of Illumina short-reads, and 63.26 Gb (90.19-fold coverage) of Hi-C sequencing data. In the process of Hi-C-assisted genome assembly, the original contigs were sorted according to the interaction map and 98.16% of the contig length could be mapped to the chromosome. Finally, we obtained an optimized assembly of 698.20 Mb with 22 chromosomes and 313 scaffolds. The contig N50 length was 796.64 kb and the scaffold N50 length was 1.09 Mb (Figure 1B; Table 1).

**Table 1:** Assembly and annotation characteristics of the *Pohlia nutans* genome.

*Genome assembly features*	
Genome size (Mb)	698.20
Max scaffold length (bp)	52,153,166
Scaffold N50 (bp)	30,709,574
Total contig length (bp)	752,686,138
Max contig length (bp)	26,973,851
Contig N50 (bp)	13,886,535
GC ratio	41.53%
*Genome annotation features*	
Number of protein coding genes	40,905
Gene density (genes/100 kb)	5.90
Average gene or CDS length (bp)	5,021/1,353
Average exons per gene	6.28
Average exon length (bp)	378.83
Average intron length (bp)	500.57
Repeat content	65.53%

A total of 40,905 protein-coding genes were identified by combining three annotation strategies, with an average coding-sequence length of 1,353.45 bp and an average of 6.28 exons per gene (Table 1). *Pohlia nutans* possessed the largest genome size and maximum gene number among the published bryophyte genomes (Figure 1C). About 93.95% of the protein-coding genes had their best homologs on plant sequences from NCBI non-redundant database, and 94.71% were functionally annotated through Swissprot, TrEMBL, Pfam, GO and KEGG databases. The genome assembly captured 86.0% of the Benchmarking Universal Single-Copy Orthologs (BUSCO) plantae dataset with 83.9% complete gene models plus 2.1% fragmented gene models (Supplementary Table 1). To assess assembly accuracy, we mapped sequencing reads to the assembled genome, with mapping rates of 97.67 and 98.86% for the paired-end and long reads, respectively (Supplementary Table 2). In addition, the Illumina paired-end reads from transcriptome data were used to evaluate assembly accuracy, receiving a 96.75% mapping rate (Supplementary Figures 1A B). Besides protein-coding genes, non-coding RNA sequences were annotated in the *P. nutans* genome, including 3,740 transfer RNAs, 55 micro RNAs, 545 ribosomal RNAs, 999 small nuclear RNAs (Supplementary Table 3). These data showed that the integrity and correctness of the *P. nutans* genome assembly were high, guaranteeing the reliability of following genomic analyses.

**Figure 2:**
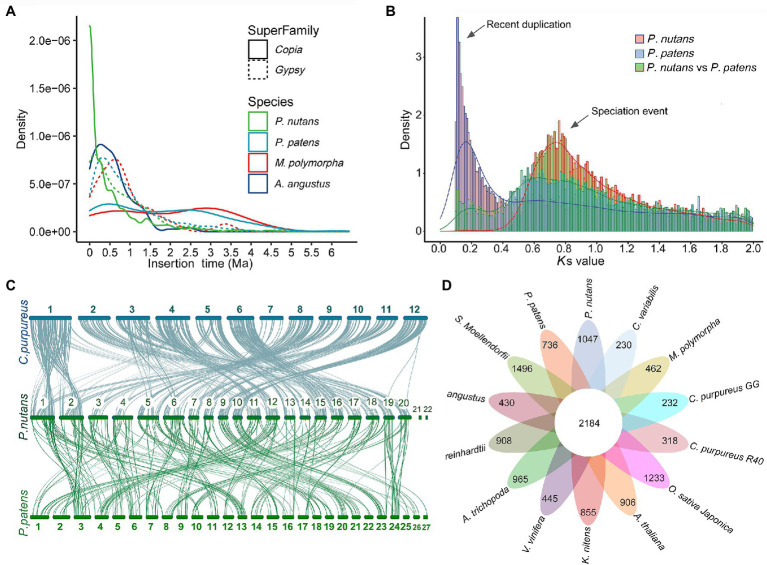
Evolutionary and comparative genome analyses of the Antarctic moss *Pohlia nutans*. **(A)** LTR-RT (*Copia* and *Gypsy*) amplification analyses of four bryophytes (the Antarctic moss *P. nutans*, the moss model plant *Physcomitrella patens*, the liverwort model plant *Marchantia polymorpha*, the hornwort model plant *Anthoceros angustus*). **(B)** Whole-genome duplication and speciation events identified between *P. nutans* and *P. patens*. **(C)** The intergenomic synteny analysis between the genomes *P. nutans*, *Ceratodon purpureus*, and *P. patens*. **(D)** Comparison of the number of gene families identified by OrthoMCL. Several representative plants were selected for evolutionary analysis such as bryophytes (*P. nutans*, *P. patens*, *C. purpureus*, *M. polymorpha*, and *A. angustus*), algae (*Chlamydomonas reinhardtii*, *Chlorella variabilis*, and *Klebsormidium nitens*), fern (*Selaginella Moellendorfii*), monocots (*Oryza sativa* and *Vitis vinifera*), eudicots (*Arabidopsis thaliana*, *A. trichopoda*, and *Clematoclethra variabilis*). The Venn diagram shows the shared and unique gene families in green plants of different evolutionary status.

We annotated repeat sequences after the genome assembly using multiple methods. Repeat sequences comprised 65.53% of the *P. nutans* assembled genome. The transposable elements (TEs) occupied 63.79% of the *P. nutans* genome assembly (Supplementary Table 4). Among the TEs annotation, long terminal repeats (LTRs) accounted for 28.77% of the genome assembly, while DNA transposons occupied 23.68%. The number of LTR-RTs in *P. nutans* genome was about 3.18-fold greater than that in *P. patens* genome (Supplementary Figure 2A). The superfamilies *Copia* and *Gypsy* were two main types of LTR-RTs, and they each accounted for 9.67 and 8.95%, respectively, of the *P. nutans* genome assembly. Particularly, *P. nutans* and hornworts (*A. angustus*) held a relative higher proportion of *Copia* type of LTR-RTs, whereas *P. patens* and liverworts (*M. polymorpha*) possessed more *Gypsy* type of LTR-RTs (Supplementary Figure 2B). *Pohlia nutans* underwent a most recent LTR-RT (*Copia* and *Gypsy*) amplification, followed by *P. patens*, *A. angustus*, and *M. polymorpha* in order (Figure 2A). Nevertheless, *Gypsy* comprised merely 1.64% of *A. angustus* genome sequences that it did not show in the plot. The density decreased between 1.0 to 6.0 Ma, which reflected element deterioration that was hard to discriminate these elements. These results indicated that LTR-RT amplification were likely contributed to the *P. nutans* genome size expansion.

Land plants normally harbor genomic signatures of whole-genome duplications (WGDs; that is, polyploidy). In order to investigate the potential WGD event, we detected the distributions of synonymous substitutions per synonymous site (*K*s) values for paralogs to identify the amplification of duplicate genes from WGDs. This method was under the assumption that synonymous substitutions between duplicate genes accrue at a relatively constant rate. Consequently, *K*s distribution of the detected pairs formed one peaks (*K*s = 0.11) demonstrating that the *P. nutans* genome presented the evidence of having undergone a recent WGD incident. Particularly, the WGD events of the *P. nutans* genome occurred more recently about at 5.85 Ma (Figure 2B). Thus, we suggested that WGD incident was another driving force for genome size expansion of *P. nutans* even more than TEs amplification.

### Comparative Genome Analysis

To evaluate the gene conservation or loss after polyploidization or speciation, we conducted the alignment of the homology of pairs of genes in the chromosomal fragments “synteny blocks.” We investigated syntenic blocks within the *P. nutans* genome or between *P. nutans* and other moss genomes using all-versus-all BLASTP alignments. The synteny analysis within the *P. nutans* genome detected a total of 1,289 syntenic blocks with 11,030 gene pairs. Generally, two adjacent chromosomes in *P. nutans* genome showed strong syntenic relationships (Supplementary Figure 2C). The data supported the fact that the *P. nutans* genome undergo a recent WGD event. The intergenomic synteny analysis between the genomes of *P. nutans* and the moss *C. purpureus* identified a total of 1,565 syntenic blocks with 16,852 gene pairs. Almost each of the *C. purpureus* chromosomes was highly syntenic with a pair of the *P. nutans* chromosomes (Figure 2C). Several exceptions were that chr7 or chr10 in *C. purpureus* exhibited syntenies with the adjacent chr7 and chr8 in the *P. nutans*, respectively; chr1 or chr11 in *C. purpureus* corresponds to the adjacent chr1 and chr2 in *P. nutans*, respectively. Meanwhile, the intergenomic synteny analysis between the genomes *P. nutans* and *P. patens* showed that there were relatively less syntenic blocks (i.e., 1,299) and gene pairs (i.e., 10,493; Figure 2C). Thus, the intergenomic syntenies between the genomes *P. nutans* and *C. purpureus* were stronger than those between the genomes of *P. nutans* and *P. patens*. In addition, the syntenic blocks between the genomes of *P. nutans* and *C. purpureus* were not congruent with those between the genomes *P. nutans* and *P. patens* (Supplementary Figure 2D).

**Figure 3:**
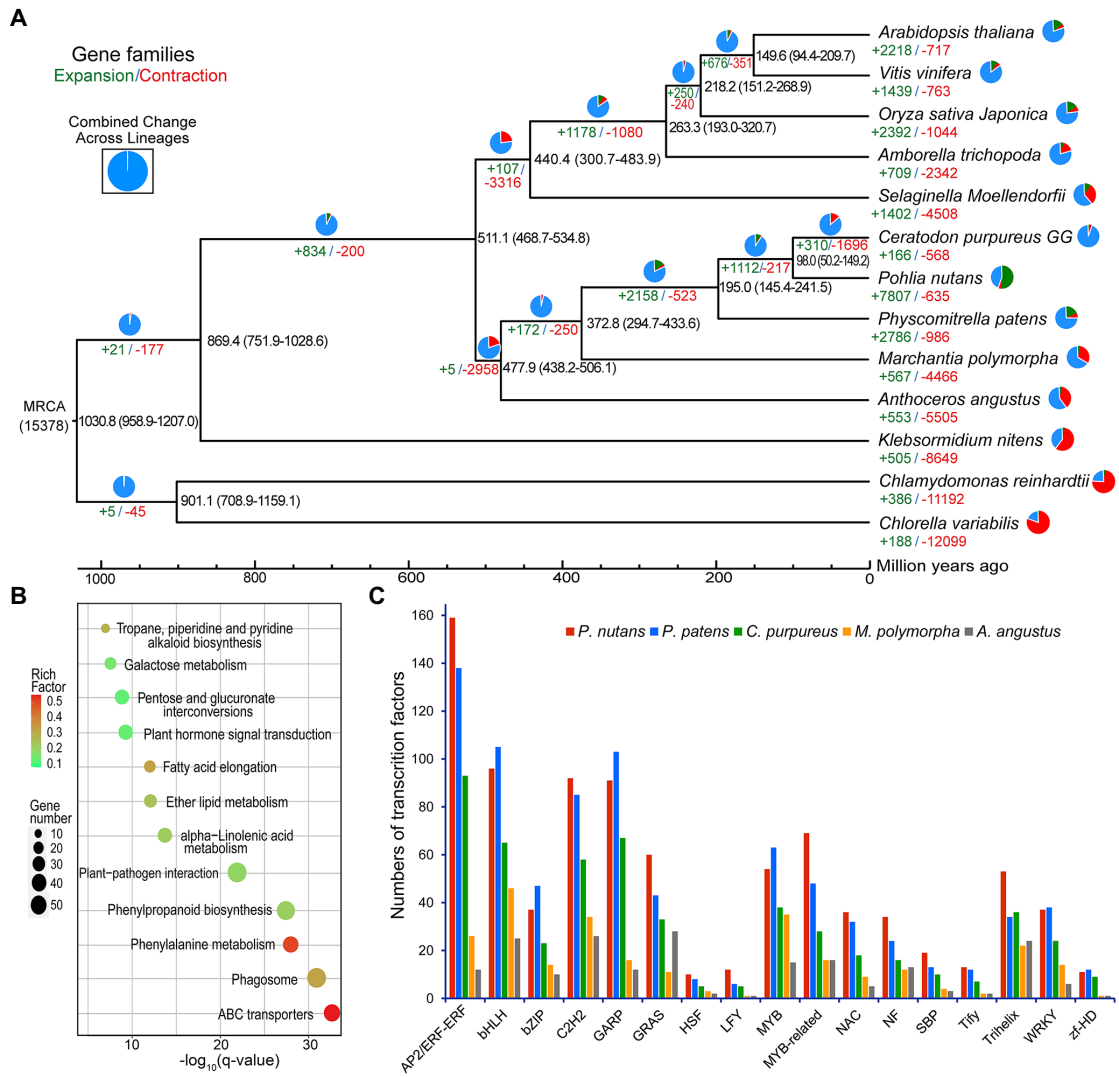
Evolutionary analysis and gene-family expansion and contraction among several green plants. **(A)** The number of significantly expanded and contracted gene families was shown on a time-calibrated phylogenetic tree. The numbers of expanded (green) and contracted (red) gene families were identified by CAFE software and shown above the branches. The estimated age of speciation was indicated on the branches with blue numbers (million years ago, Ma). **(B)** Scatterplot of KEGG enrichment of the expanded gene families in *Pohlia nutans*. **(C)** Numbers of selected stress-related transcription factor genes in five bryophyte species.

For sequence similarity-based clustering of homologues, we conducted the analyses with the predicted proteomes among *P. nutans* and other 13 green plants. These representative green plants included bryophytes (*P. patens*, *C. purpureus*, *M. polymorpha*, and *A. angustus*), algae (*Chlamydomonas reinhardtii*, *Chlorella variabilis*, and *Klebsormidium nitens*), fern (*Selaginella Moellendorfii*), monocots (*Oryza sativa* and *Vitis vinifera*), and eudicots (*Arabidopsis thaliana*, *A. trichopoda*, and *Clematoclethra variabilis*). We identified 37,125 genes distributed among 12,650 gene families in *P. nutans*. A total of 5,357 gene families were shared by four species *P. nutans*, *P. patens*, *A. angustus*, and *K. nitens* genomes, while a total of 3,523 gene families collectively presented in above four species and *C. reinhardtii* (Supplementary Figures 3A B). In addition, 2,184 gene families were shared by 14 represent green plants with different evolutionary status, whereas 1,047 gene families including 2,790 genes, which appeared to be unique to *P. nutans* (Figure 2D). These lineage-specific family genes were markedly enriched in various biosynthetic categories (e.g., phenylpropanoid biosynthesis, flavonoid biosynthesis, carotenoid biosynthesis, and other biosynthesis), likely facilitating neofunctionalization (Supplementary Figure 3C). *Pohlia nutans* genome possessed the largest number of protein-coding genes, but few amount of single-copy orthologs (Supplementary Figure 3D). We therefore use half of the paired chromosomes of *P. nutans* for species evolutionary analyses. Finally, the phylogenetic position of *P. nutans* was determined using 51 single-copy orthologs (Figure 3A). *Pohlia nutans* was clustered within bryophyte lineages, sister to the temperate *C. purpureus* species. Our molecular dating analyses indicated that the speciation of bryophyte lineages occurred at 511.1 Ma and the divergent event between *P. nutans* and *C. purpureus* occurred at 98.0 Ma (Figure 3A).

We assessed the gene family expansion or contraction by using the homolog matrix of orthogroups to calculate ancestral and lineage-specific genes on the phylogenetic tree. The genome of *P. nutans* covered 7,807 markedly expanded gene families and 635 notably contracted gene families (*p* < 0.05), respectively (Figure 3A). Particularly, *P. nutans* harbored the largest number of expanded gene families among 14 green plants. Furthermore, KEGG pathway enrichment analyses revealed that the expanded gene families participated in biosynthesis of metabolites (i.e., phenylpropanoid biosynthesis, tropane, piperidine, and pyridine alkaloid biosynthesis, alpha-Linolenic acid metabolism, and fatty acid elongation), environmental adaptation (i.e., plant hormone signal transduction, and plant-pathogen interaction; Figure 3B).

The *P. nutans* genome comprised of 1,328 transcription factors (TFs) forming 63 families, which was substantially larger than these in other four bryophyte genomes (Figure 3C). In addition, the *P. nutans* genome also harbored more stress-related TFs like AP2, bHLH, bZIP, MYB, NAC, Trihelix, and WRKY (Figure 3C).

### Evolutionary Innovations of Stress-Related Genes in Extreme Terrestrial Habitats

**Figure 4:**
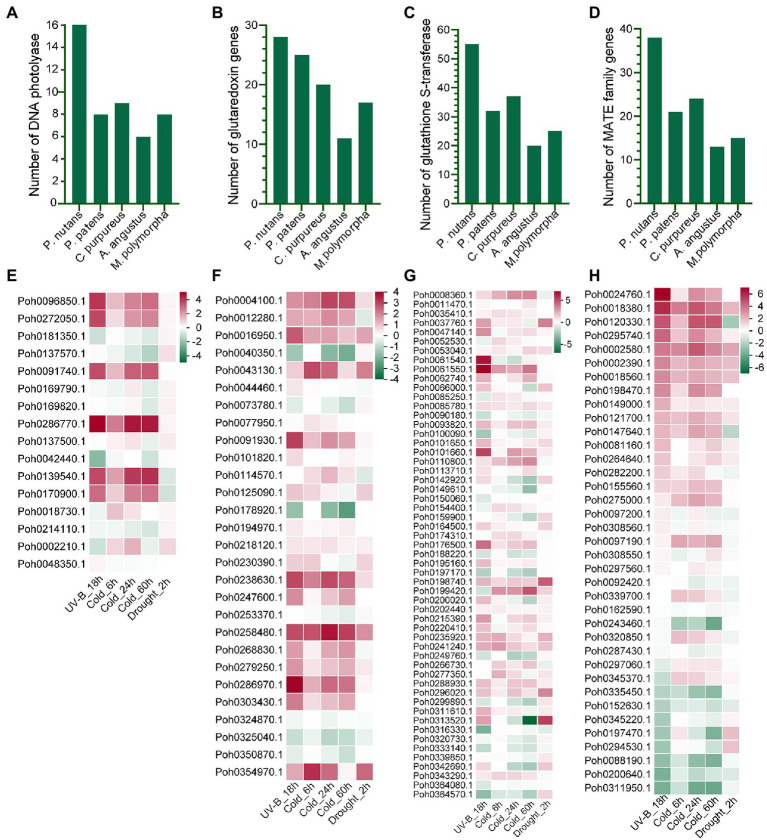
The gene families related to oxidative stress were expanded and upregulated under various abiotic stresses. Gene families of DNA photolyase **(A)**, glutaredoxin **(B)**, glutathione S-transferase **(C)**, and protein detoxification with of MATE domain **(D)** were expanded in the *P. nutans* genome. Genes encoding DNA photolyase **(E)**, glutaredoxin **(F)**, glutathione S-transferase **(G)**, and protein detoxification with MATE domain **(H)** were markedly upregulated under UV-B radiation, cold, and drought stresses detected by transcriptome sequencing.

Although the Antarctic Peninsula is warming and the East Antarctica is likely to become dryness, it is obviously that strong ultraviolet radiation is the most significant adverse result of climate change and human activities. Therefore, we will focus on the molecular mechanism of *P. nutans* in response to strong UV-B radiation. We found that the gene family of DNA photolyase was highly expended in *P. nutans* when compared with other four bryophytes. There were 16 of DNA photolyase homologs in the *P. nutans* genome, nine in the *C. purpureus* genome, eight in the *P. patens* genome, eight in the *M. polymorpha* genome, and six in the *A. angustus* genome (Figure 4A). In addition, we performed the transcriptome sequencing of *P. nutans* under UV-B radiation, cold stress, and drought stress. Six DNA photolyase genes were significantly upregulated under UV-B radiation and cold stress (Figure 4E). We also found that gene families encoding antioxidant and detoxicant enzymes were expanded in *P. nutans*, such as glutaredoxin, glutathione S-transferase, and protein detoxification of MATE family (Figures 4C,D). They were also differentially expressed under UV-B radiation, cold and drought stresses (Figures 4F–H). Thus, the DNA photolyases and antioxidant enzymes could provide the Antarctic moss *P. nutans* with DNA repair machinery and ROS-scavenging system against environmental stresses.

### Integrated Transcriptomic and Metabolomic Analysis Highlight the Role of Flavonoid Biosynthesis Under UV-B Light

**Figure 5:**
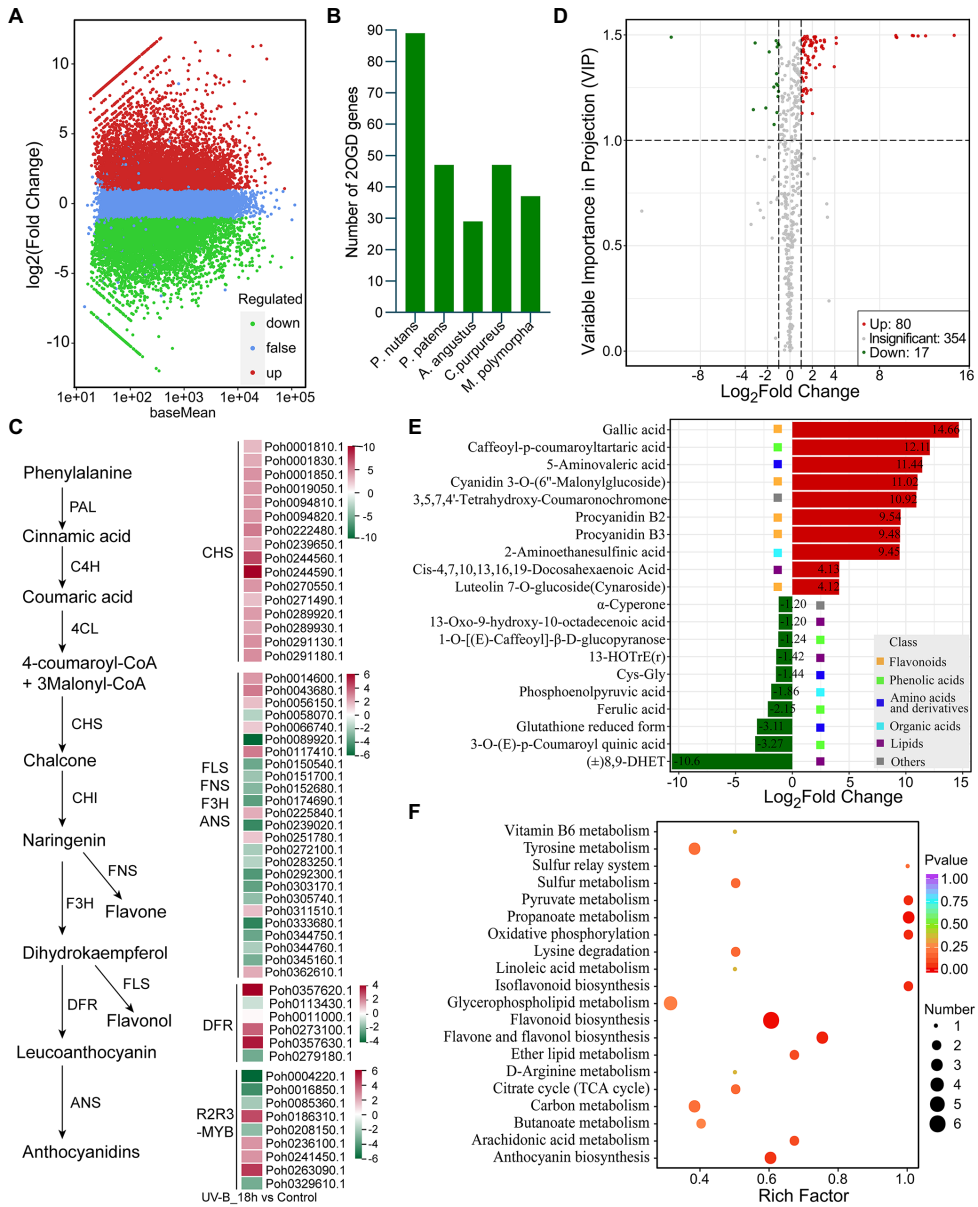
Integrated multi-omic analyses reveal the role of flavonoid pathway under UV-B light. **(A)** The volcano plot showing the DEGs between UV-B radiation group and control group. The *Y*-axis indicates fold change of gene expression (threshold, |log_2_(Treat/Control)| > 1), while the *X*-axis means the statistically significant level (threshold, *q*-value < 0.05). **(B)** The gene family of 2-oxoglutarate-dependent dioxygenase (2-OGD) was expanded in the *Pohlia nutans* genome. **(C)** Flavonoid biosynthesis pathway genes were markedly upregulated under UV-B radiation detected by transcriptome sequencing. **(D)** The volcano plot showing the contents of metabolites and the statistical significance. Each point represents a metabolite. Horizontal ordinate indicates the fold change of metabolites between two groups, while VIP value represents significant difference in statistical analysis. **(E)** The fold change of the top 20 significantly changed metabolites (SCMs) between two groups. **(F)** Statistics of KEGG enrichment for the SCMs.

We found the gene family encoding phenylalanine ammonia-lyase (PAL), catalyzing the first committed step of the phenylpropanoid pathway, was highly expanded in *P. nutans* genome. Phylogenetic analysis showed that most of PAL homologs from mosses were clustered together (Supplementary Figure 4A). Two PAL homologs from hornwort and four moss PAL homologs were close to the tree root, which seemed to be more ancestral. In addition, we performed the transcriptome sequencing of *P. nutans* under UV-B radiation (Figure 5A). We found that 14 PAL genes were significantly upregulated in *P. nutans* under UV-B radiation (Supplementary Figure 4B). The flavonoid biosynthesis enzymes were expanded in the *P. nutans* genome, including chalcone synthase (CHS) and 2-oxoglutarate-dependent dioxygenase (2-OGD; Figure 5B). 2-OGD family proteins like flavonol synthase, flavone synthase, flavanone-3-hydroxylase, and anthocyanidin synthase harbored the conserved domains of 2OG-FeII_Oxy and DIOX_N. Transcriptome sequencing showed that *CHS* and *2-OGD* were markedly upregulated under UV-B radiation. In higher plants, the transcription factor R2R3-MYB is responsible for regulating the transcription of flavonoid biosynthesis enzymes (Albert et al., 2018). Transcriptome sequencing also showed that six *R2R3-MYB* were markedly upregulated in *P. nutans* under UV-B radiation (Figure 5C).

To further reveal the responses of *P. nutans* to UV-B radiation, we used the UPLC-MS/MS method to detect the metabolites. A total of 415 metabolites were identified (Supplementary Table 5). Flavonoids accounted for 9.88% of the total compounds. In addition, anthocyanins were also identified in *P. nutans*, including cyanidin 3-O-(6″-Malonylglucoside), malvidin 3-O-galactoside, and peonidin O-hexoside. PCA score plot, heatmap cluster, and the supervised OPLS-DA analyses indicated that the data were stable and reliable, and significant differences were detected in metabolic phenotypes after UV-B radiation (Supplementary Figures 5A C). Using thresholds of |log_2_Foldchange| ≥ 1 and VIP (variable importance in project, VIP) ≥ 1, a total of 97 significantly changed metabolites (SCMs) were detected under UV-B radiation. Of them, 80 metabolites were significantly upregulated and 17 metabolites were markedly downregulated (Figure 5D; Supplementary Table 6). The top 20 SCMs in UV-B group compared to control group according to the values of log_2_(Fold change) are shown in Figure 5E.

Flavonoids accounted for 37.11% of the total SCMs (Supplementary Figure 5D). Gallic acid was the most accumulated metabolite with log_2_(Fold change) 14.66 and VIP score 1.50. Caffeoyl-p-coumaroyltartaric acid, a kind of phenolic acids, was the second significantly changed metabolite with log_2_(Fold change) 12.11 and VIP score 1.50. In addition, cyanidin 3-O-(6″-malonylglucoside), a kind of anthocyanins, was also markedly accumulated metabolite with log_2_(Fold change) 11.02 and VIP score 1.5, exhibiting significant antioxidant activity (Rahman et al., 2021). KEGG pathway analysis showed that the SCMs were mainly involved in flavonoid biosynthesis pathways (Figure 5F). Notably, DEGs and SCMs were both abundantly enriched in flavonoid biosynthesis pathway under UV-B radiation. These metabolites might facilitate mosses in resisting the extra ROS damages. Therefore, the gene-family expansions, upregulated mRNA levels, and accumulated metabolites in flavonoid biosynthesis pathway might represent one of molecular adaptations to life in the Antarctic strong UV-B environments.

## Discussion

### The Antarctic Moss Genome Provides New Resource for Evolutionary and Functional Studies

Antarctic extremely environmental pressures including higher UV-B radiation, freezing, and extreme dryness, lower nutrient supply and strong wind severely influence the reproduction and distribution of terrestrial plants (Convey and Peck, 2019; Zhang et al., 2020b). Mosses and lichens dominate the Antarctic flora and are restricted to sparse ice-free areas (Skotnicki et al., 2002). They are normally at the survival limitations. These native terrestrial plants have adapted to the extreme conditions over many millions of years (Convey and Peck, 2019). They are now threatened by global climate changes and the direct impacts of human activities (Malenovský et al., 2015; Amesbury et al., 2017; Robinson et al., 2018), as well as invasions of non-native species (Hughes et al., 2020). Here, we complete a high-quality genome sequence of the Antarctic moss *P. nutans* with 699.88 Mb with 22 chromosomes and 362 scaffolds (Figure 1). The distribution of *K*-mer frequency was widely used for the estimation of genome size (Zhang et al., 2020b). The optimized genome assembly was largely congruent with the *K*-mer analysis. The genomes of representative species such as mosses (*P. patens* and *Syntrichia caninervis*), liverworts (*M. polymorpha*), and hornworts (*A. angustus*) have been sequenced and offer new perspectives for understanding the molecular adaptations to terrestrial life (Bowman et al., 2017; Lang et al., 2018; Zhang et al., 2020a; Silva et al., 2021).

Compared with the genome size of other bryophytes, the *P. nutans* genome was substantially larger with a higher proportion of LTR-RTs and more protein encoding genes (Figure 1C; Table 1). Repeat sequences comprised 65.53% of the *P. nutans* assembled autosomal genome (Table 1; Supplementary Table 4), similar to that of *A. angustus* (64.21%; Zhang et al., 2020a) and the Antarctic ice algae ICE-L (63.78%; Zhang et al., 2020b), but a remarkable higher than that of *M. polymorpha* (22%; Bowman et al., 2017) and *P. patens* (48%; Rensing et al., 2008). TEs comprised 63.79% of the *P. nutans* genome assembly, whereas they accounted for 57% of the *P. patens* genome (Lang et al., 2018). Notably, LTR-RTs in *P. nutans* genome was the most abundant among the four bryophyte genomes (Figure 2A; Supplementary Figure 2A). We therefore proposed that the amplification of LTR-RTs in *P. nutans* likely enlarges its genome size and may play an essential role in the adaptations to extreme environments.

High proportion of repetitive sequences usually causes a large barrier to reliable assembly of genomes from short-read sequences (Uauy, 2017). Consequently, the *P. nutans* genome assembly captured 86.0% of the BUSCO plantae dataset with 83.9% complete gene models plus 2.1% fragmented gene models (Manni et al., 2021), compared with 89.64, 93.51, and 92.15% captured in *A. angustus* (Zhang et al., 2020a), *P. patens* (Rensing et al., 2008; Lang et al., 2018), and *M. polymorpha* (Bowman et al., 2017), respectively (Supplementary Table 1). Similarly, in Antarctic green alga *Chlamydomonas* sp. ICE-L, the genome assembly captured 83.7% of the BUSCO datasets (Zhang et al., 2020b). In Antarctic green alga *Chlamydomonas* sp. UWO241, the genome assembly contains 16,325 protein-coding genes, capturing 85% of the BUSCO datasets (Zhang et al., 2021). We speculate that organisms living in extreme conditions generated multiple new metabolic pathways and functional genes, but that several metabolic pathways are missing, resulting in a lower BUSCO score.

Bryophytes comprise of mosses, hornworts, and liverworts that are the three early diverging extant land plant lineages (Bowman et al., 2017). Given that they emerged from the early lineage in the divergence of land plants, they offer the critical clues to investigate the early land plant evolution (Zhang et al., 2020a). Thus, this high-quality assembled genome will provide an important resource for evolutionary and functional studies in mosses, particularly those associated with UV-B radiation, cold, and drought resistance studies.

### The Recent WGD Was a Major Fountain for Gene-Family Expansion and Function Diversification

Whole-genome duplication incident is an important driving force for speciation, with approximately 15% of angiosperm and 31% of fern speciation events involving this process (Wood et al., 2009; Hong et al., 2021). In moss model plant *P. patens*, *K*s-based analysis indicated two WGD incidents dating back to 27–35 and 40–48 Ma, respectively (Lang et al., 2018). Like that of *P. patens*, the *P. nutans* genome presented the evidence of having undergone a WGD event, which occurred markedly earlier than that of *P. patens* (Figure 2B). In *P. patens* genome, the *K*s values of the structure-based peaks are between 0.75–0.90 (older WGD1) and 0.50–0.65 (younger WGD2; Lang et al., 2018). *K*s frequency analysis between *P. nutans* and *P. patens* identified a notable *K*s peak at about 0.75 of duplicated syntenic pairs, indicating that the older WGD1 event was ancestrally shared by the two genomes and subsequently the speciation event was occurred (Figure 2B). The syntenic blocks within the *P. nutans* genome showed that most of two adjacent chromosomes had strong syntenic relationships (Figure 1B; Supplementary Figure 2C). In haploid *P. nutans*, chromosome number *n* = 11, 22, 33 are the most common and have been recorded from many countries, including Estonia (*n* = 11; Fetisova and Visotskaya, 1970), Australia (*n* = 22; Ramsay and Spence, 1996), and British and Irish (Smith and Newton, 1967). Given the syntenic relationships (Supplementary Figure 2C), one of the most likely assumptions for the extant chromosome number of the Antarctic moss *P. nutans* was the duplication of 11 ancestral chromosomes by WGD event, followed by chromosomal rearrangement, and DNA segment exchange, fusion and loss, as well as other fragmentation events. Finally, the genome of Antarctic moss *P. nutans* formed 22 chromosomes. This genome also showed high synteny with the previously released genomes of *P. patens* and *C. purpureus* (Figure 2C). Interestingly, almost each chromosome in *C. purpureus* was syntenic with two adjacent chromosomes in *P. nutans*. Thus, we inferred that the *P. nutans* genome was forming haploidy after undergoing a recent WGD event and still retained a large number of duplicated blocks within its genome.

Whole-genome duplication also represents one of the primary mechanisms for gene duplication, along with three principal evolutionary patterns such as tandem duplication, segmental duplication, and transposition events (Hong et al., 2021). In addition, gene duplicates comprise 8–20% of the genes in eukaryotic genomes, which facilitates the establishment of new gene functions and the generation of evolutionary novelty (Moore and Purugganan, 2003; Van De Peer et al., 2009). Segmental and tandem duplications are two predominate causes for gene family expansion in plants. We identified 28,529 segmental duplication gene pairs in *P. nutans* and accounted for 76.29% of the total duplication gene pairs. Segmental duplications multiple genes are generated through the chromosome rearrangements following WGD (Hong et al., 2021). Thus, the high proportion of segmental duplication gene pairs was largely congruent with the above analysis that *P. nutans* had undergone a recent WGD event (Figure 2B). Tandem duplications are defined as multiple genes of one family emerging within the same intergenic region or in neighboring intergenic regions. In absence of WGD in *A. angustus*, the tandem gene duplications mainly contribute to the expansion of specialized gene family and the adaptive evolution of the hornwort during the colonization of terrestrial ecosystems (Zhang et al., 2020a).

Some central gene-family expansions continuously occurred after the origin of embryophytes, especially in the origin of land plants and the evolution of bryophytes (Leebens-Mack et al., 2019). WGD provides a major fountain for large expansions of gene families. Through WGD events, the duplication of all genes could offer an extraordinary opportunity for the occurrence of evolutionary novelties and functional diversifications (Van De Peer et al., 2009; Hong et al., 2021). These mechanisms may support adaptive evolution and have likely contributed plant survival in terrestrial habitats. Accordingly, we identified 7,807 of significantly expanded and 635 of markedly contracted gene families in the *P. nutans* genome (Figure 3A). The origin of land plants and the evolution of bryophytes accompany the largest number of gene-family expansions (i.e., transition from streptophyte algae to bryophytes; Leebens-Mack et al., 2019). Notably, in the *P. nutans* genome, the number of expanded gene families was remarkable large, being even 2.80-fold than that in the *P. patens* genome (Figure 3A). TFs-encoding genes were among the most highly retained following WGD incident, which exhibited in the present comparison of five bryophyte genomes. Since *P. nutans* and *P. patens* genomes both underwent at least one WGDs that they possessed a large number of TFs, whereas *A. angustus* and *M. polymorpha* genomes did not experience WGDs holding far less TFs. Particularly, several TFs like AP2, bHLH, bZIP, and WRKY emerged in the latest ancestor of Viridiplantae, whereas GRAS and NAC family genes arose in early streptophytes after divergence from the chlorophytes (Leebens-Mack et al., 2019). We found that they all emerged in these five bryophyte genomes (Figure 3C).

The gene families encoding DNA photolyase, antioxidant enzymes, PAL, CHS, and 2-OGD were significantly upregulated under various abiotic stresses, possibly representing diverse adaptive processes (Figures 4, 5). Photolyase is responsible for repairing the UV-induced DNA damage in a light-dependent manner. We found that the gene family of DNA photolyase was highly expended in *P. nutans* when compared with other four bryophytes (Figure 4A). Previously, we reported a CPD photolyase gene *PnPHR1* isolated from Antarctic moss *P. nutans*. PnPHR1 can repair photoproducts of cyclobutane pyrimidine dimers (CPD) and enhance the plant resistance to UV-B radiation by scavenging extra ROS (Wang et al., 2021a). PnPHR1 also increases the survival rate of *Escherichia coli* strain after UV-B radiation. Therefore, the successful colonization of *P. nutans* in Antarctic extreme environments can be attributed to the large expansion of gene families with functions associated with DNA repairing machine, ROS scavenging system, unsaturated fatty acid biosynthesis, and flavonoid biosynthesis.

### Flavonoid Metabolites Play an Important Role in UV-B Tolerance of *Pohlia nutans*

Antarctic terrestrial organisms have undergone some of the most extremely environmental pressures including higher UV irradiation, cold, and extreme dryness. The production of a diverse repertoire of specialized metabolites is one of the features of land plants in response to environmental stresses (Yang et al., 2018; de Vries et al., 2021). The phenylpropanoid pathway is the sources for lots of metabolites that act in warding off environmental stressors (de Vries et al., 2021). The phenylpropanoid and flavonoid pathways likely facilitate the earliest steps of plants on land, protecting pioneer plants against the threatens of the terrestrial stresses such as drought and increased UV radiation (Jiao et al., 2020; de Vries et al., 2021). While some of flavonoids are the well-known UV screening compounds, various other phenylpropanoid-derived metabolites are equally potent UV protectants (Zeng et al., 2020; Nichelmann and Pescheck, 2021). We found that PAL gene family was markedly expanded in *P. nutans* when compared with other four bryophytes (Supplementary Figure 4). Of them, 14 PAL genes were markedly upregulated in *P. nutans* under UV-B radiation. Some phenylpropanoids undertake a screening barrier increasing resistance to UV-B light in the Antarctic moss *C. purpureus* (Clarke and Robinson, 2008). Flavonoids are produced through the phenylpropanoid and acetate-malonate metabolic pathways (Buer et al., 2010). They are deemed to have emerged during plant landing and adapting to terrestrial habitats about 500 million years ago (Davies et al., 2020; Stiller et al., 2021). Bryophytes and angiosperms likely have both commonalities and significant differences in flavonoid biosynthesis and metabolic regulation (Davies et al., 2020). We found that enzymes of flavonoid pathway were expanded in *P. nutans*, such as CHS and 2-OGD (Figure 5B). In addition, some of these genes were upregulated under UV-B radiation (Figure 5C). Previously, we characterized a type I flavone synthase (i.e., PnFNSI) from *P. nutans*. PnFNSI could catalyze the conversion of naringenin to apigenin and increase plant tolerance to UV-B radiation and drought stress (Wang et al., 2020). Similarly, flavonoids and carotenoids extracted from three Antarctic moss species demonstrate the features of UV-absorbing compounds and activate the DNA damage repair process (Pereira et al., 2009).

Gene-family expansions or contractions contribute to the production of a diverse repertoire of specialized metabolites in response to environmental stresses (Leebens-Mack et al., 2019; de Vries et al., 2021). However, analytical approaches in earlier publications largely use the simple equipment of spectrophotometer (i.e., UV-B-absorbing compounds at AUC_280–315 nm_, anthocyanins at A_526 nm_; Waterman et al., 2018). The HPLC-MS/MS-based plant metabolomics provides a qualitative and quantitative solution and has been extensively used to profile diverse repertoire of specialized metabolites (Li and Song, 2019; Wang et al., 2021b). Here, a widely targeted metabolomics built on the UPLC-MS/MS platforms found that 80 metabolites were markedly upregulated, and 17 metabolites were notably downregulated in *P. nutans* after UV-B stress (Figure 5D). KEGG enrichment indicated that these SCMs were mainly involved in flavonoid biosynthesis, flavone and flavonol biosynthesis, anthocyanin biosynthesis, and isoflavonoid biosynthesis and other small molecule metabolites (Figure 5F). Among them, gallic acid (a kind of flavonol) was the most significantly changed metabolite of with log_2_Fold change 14.66. Particularly, abiotic stress usually causes the generation of ROS and oxidative damage. Plants generally maintains a delicate balance of ROS through efficient ROS scavenging system. Gallic acid serves a natural defense mechanism by exhibiting excellent antioxidant and improving leaf membrane stability (Choubey et al., 2018; Saidi et al., 2021). In addition, cyanidin 3-O-(6″-malonylglucoside), a kind of anthocyanins, was also significantly accumulated with log_2_(Fold change) 11.02 (Supplementary Table 6), exhibiting predominant antioxidant effects against various oxidative stress (Rahman et al., 2021). In contrast, anthocyanins were not detected in methanolic extracts of *P. patens* separated by HPLC method (Wolf et al., 2010). Similarly, several SCMs like procyanidin and luteolin were detected in *P. nutans*, and functioned as an efficient antioxidant (Gong et al., 2020). Notably, several Antarctic mosses (e.g., *Bryum pseudotriquetrum*, *C. perpureus*, and *Schistidium antarctici*) and liverwort (*M. polymorpha*) possess anthocyanins, but Antarctic algae do not have anthocyanins (Singh et al., 2011). Flavonoids will reduce the transmittance of UV-B light and enabled plants to first colonize land (Singh et al., 2012; Singh and Singh, 2014). Therefore, transcriptomics integrated with metabolomics offer evidence for the opinion that flavonoids act as efficient antioxidants might dominate the tolerance of *P. nutans* against UV-B radiation.

## Conclusion

Mosses are the basal land plants thriving in Antarctic ice-free continent and have evolved to survive, grow, and propagate in this harsh environment. We found that the Antarctic moss *P. nutans* genome harbors the signatures of a recent WGD incident with a high proportion of repeat sequences. In particular, the massive segmental gene duplications and remarkable expansions of gene families provide the primary driving force for the evolution of novel gene function and the production of specialized metabolite repertoire. Notably, the integration of multi-omics data elucidates the underlining mechanism of Antarctic moss *P. nutans* adaptation to extreme environments, which can be largely attributed to DNA repairing, ROS scavenging, and flavonoid biosynthesis. Collectively, the high-quality genome of *P. nutans* provides insight into the unique features of early land plant evolution and the molecular mechanism of moss surviving under rapidly changed environments in Antarctica.

## Data Availability Statement

The plant material is vouchered and available on request from Dr. Linlin Zhao at the First Institute of Oceanography, Natural Resources Ministry of China. The whole genome sequence data presented in the study are deposited in the National Genomics Data Center (NGDC, https://ngdc.cncb.ac.cn), BioProject number PRJCA008231. Of them, the PacBio HiFi reads of *P. nutans* were deposited in Genome Sequence Archive (GSA), accession number CRA006048; the Hi-C sequencing reads were deposited in GSA under the accession number CRA006049; the assembly and annotation data were deposited in Genome Warehouse (GWH), accession number GWHBHNB00000000. In addition, the transcriptome sequencing data of *P. nutans* were also deposited in GSA, accession number CRA006053, CRA006553, and CRA006556.

## Author Contributions

LZ and PZ conceived and designed the project and revised the manuscript. SL prepared the samples and performed the analyses of the genome, transcriptome sequence, with assistance from TL. SF performed the comparative genomics. BC, DY, and ZZ performed the metabolomics profiling and analysis. SL wrote the manuscript. All authors contributed to the article and approved the submitted version.

## Funding

This research was funded by the National Natural Science Foundation of China, grant/award number: 41976225; the Central Public-Interest Scientific Institution Basal Research Foundation of China, grant/award number: GY0219Q05; and the Development Project of Shandong Province, grant/award number: 2019GSF107064.

## Conflict of Interest

The authors declare that the research was conducted in the absence of any commercial or financial relationships that could be construed as a potential conflict of interest.

## Publisher’s Note

All claims expressed in this article are solely those of the authors and do not necessarily represent those of their affiliated organizations, or those of the publisher, the editors and the reviewers. Any product that may be evaluated in this article, or claim that may be made by its manufacturer, is not guaranteed or endorsed by the publisher.

## Footnotes

^1^ http://www.timetree.org/

## Supplementary Material

Supplementary Table 1 Comparison of BUSCO assessment of genome annotation among four bryophytes.

Supplementary Table 2 Statistics of sequencing reads mapping to the Pohlia nutans genome assmebly.

Supplementary Table 3 Identification of non-coding RNA genes in the Pohlia nutans genome.

Supplementary Table 4 Summary of transposable elements (TEs) identified in the Pohlia nutans genome.

Supplementary Table 5 All detected metabolites in Pohlia nutans under UV-B Radiation.

Supplementary Table 6 Significantly changed metabolites in Pohlia nutans under UV-B radiation.

Supplementary Figure 1 Mapping analysis of transcriptome sequencing data. **(A)** The density of Illumina paired-end reads from transcriptome data in 22 chromosomes of *Pohlia nutans*. **(B)** Number of mapped reads from transcriptome sequencing in 22 chromosomes of *P. nutans*. Transcriptome sequencing data from cold stress were used for this assessment. Fix layout: justify the text.

Supplementary Figure 2 LTR-RT evolution and chromosome syntenic analyses. **(A)** Number of intact LTR-RTs of *Pohlia nutans*, *Physcomitrella patens*, *Marchantia polymorpha*, and *Anthoceros angustus*. **(B)** Phylogenetic relationships of *Copia* and *Gypsy* retrotransposons across four bryophytes. **(C)** Dotplot representation of the introgenomic synteny analysis within the *P. nutans* genome. **(D)** Compare the consistency of syntenic blocks between two groups of *P. nutans* vs. *C. purpureus*, and *P. nutans* vs. *P. patens*.

Supplementary Figure 3 Venn diagram of gene families and clustering statistics. **(A)** The Venn diagram shows the shared and unique gene families in *Pohlia nutans*, *Physcomitrella patens*, *Anthoceros angustus*, and *Klebsormidium nitens* genomes. **(B)** The Venn diagram shows the shared and unique gene families in *P. nutans*, *P. patens*, *A. angustus*, *K. nitens*, and *Chlamydomonas reinhardtii* genomes. **(C)** Scatterplot of KEGG enrichment of the unique family genes in *P. nutans*. **(D)** Clustering statistics of gene family among the green plants of different evolutionary status.

Supplementary Figure 4 Phenylalanine ammonia-lyase (PAL) family was expanded and upregulated under various abiotic stresses. **(A)** Phylogenetic analysis of PAL families in five bryophyte species (*Pohlia nutans*, *Physcomitrella patens*, *Marchantia polymorpha*, and *Anthoceros angustus*). The branches of *P. nutans* were colored in red, and the gene ID of *P. nutans* were shown. **(B)** Heatmap showed that PAL genes were markedly upregulated under cold stress, and UV-B radiation detected by transcriptome sequencing.

Supplementary Figure 5 Statistical analysis of data quality and differential metabolites. **(A)** Principal component analysis (PCA) for mass spectrum data of UV-B radiation group and control group, and mix (quality control sample). The *x* axis and *y* axis indicate the PC1 and PC2, respectively. Three groups (i.e., CG, UV-B, and mix) were clearly separated, and three biological replicates of each group were clustered together, showing that the experiments were reproducible and reliable. **(B)** Heatmap shows that there were significant differences in the classes and quantities of metabolites between UV-B radiation group and control group. The green color indicates the decrease of differentially expressed metabolites, while the red color indicates the increase of differentially expressed metabolites. **(C)** Score scatter plot of the latent structure discriminant analysis (OPLS-DA) model for the differences between UV-B radiation group and control group. *R*^2^*X* and *R*^2^*Y* indicate the interpretation rate of *X* and *Y* matrix, respectively. *Q*^2^*Y* represents the prediction ability of the model, while a value closer to 1.0 means that the model is more stable and reliable. In addition, *Q*^2^*Y* > 0.5 can be regarded as an effective model, and *Q*^2^*Y* > 0.9 is an excellent model. The data indicated that UV-B radiation strongly changed the metabolite profiles of the Antarctic moss *Pohlia nutans*. **(D)** Statistics of significantly changed metabolites in *P. nutans* under UV-B Radiation.
