# Supplementary material for: The Antarctic Moss Pohlia nutans Genome Provides Insights Into the Evolution of Bryophytes and the Adaptation to Extreme Terrestrial Habitats
Source: Front Plant Sci. 2022 Jun 17;13:920138. doi: 10.3389/fpls.2022.920138 (PMC9247546; doi:10.3389/fpls.2022.920138)
Supplement: Supplementary Table 1 — Comparison of BUSCO assessment of genome annotation among four bryophytes. [file Data_Sheet_1.zip › Data Sheet 1/Table 1 (82).docx]

**Supplementary Table 1.** Comparison of BUSCO assessment of genome annotation among four bryophytes.

|  | *Pohlia nutans* | | *Anthoceros angustus* | | *Marchantia polymorpha* | | *Physcomitrella patens* | |  |
| --- | --- | --- | --- | --- | --- | --- | --- | --- | --- |
|  | Proteins | Percent (%) | Proteins | Percent (%) | Proteins | Percent (%) | Proteins | Percent (%) | |
| Complete BUSCOs | 802 | 83.9 | 813 | 85.04 | 859 | 89.85 | 841 | 87.97 |  |
| Complete Single-Copy BUSCOs | 216 | 22.6 | 529 | 55.33 | 555 | 58.05 | 462 | 48.33 |  |
| Complete Duplicated BUSCOs | 586 | 61.3 | 284 | 29.71 | 304 | 31.80 | 379 | 39.64 |  |
| Fragmented BUSCOs | 20 | 2.1 | 44 | 4.60 | 22 | 2.30 | 53 | 5.54 |  |
| Missing BUSCOs | 134 | 14 | 99 | 10.36 | 75 | 7.85 | 62 | 6.49 |  |
| Total BUSCO groups searched | 956 | 100 | 956 | 100.00 | 956 | 100.00 | 956 | 100.00 |  |
