# Supplementary material for: The Antarctic Moss Pohlia nutans Genome Provides Insights Into the Evolution of Bryophytes and the Adaptation to Extreme Terrestrial Habitats
Source: Front Plant Sci. 2022 Jun 17;13:920138. doi: 10.3389/fpls.2022.920138 (PMC9247546; doi:10.3389/fpls.2022.920138)
Supplement: Supplementary Table 1 — Comparison of BUSCO assessment of genome annotation among four bryophytes. [file Data_Sheet_1.zip › Data Sheet 1/Table 2 (37).docx]

**Supplementary Table 2**. Statistics of sequencing reads mapping to the *Pohlia nutans* genome assmebly.

| Sequencing method | Mapping rate(%) | Average sequencing depth | Coverage(%) | Coverage at least 4×(%) | Coverage at least 10×(%) | Coverage at least 20×(%) |
| --- | --- | --- | --- | --- | --- | --- |
| Illumina short-reads | 97.67 | 106.92 | 99.78 | 99.32 | 98.25 | 96.26 |
| PacBio Sequel Ⅱ HiFi-reads | 98.86 | 40.61 | 99.29 | 96.22 | 92.88 | 91.37 |

**The meaning of statistical items are as follows:**

1. Mapping rate(%), the rate of reads that can be compared to the genome.
2. Average sequencing depth, the average sequencing depth of sequencing data.
3. Coverage(%), the rate of the whole genome covered by sequencing data.
4. Coverage at least 4×(%), the rate of the whole genome covered by at least 4×sequencing data.
5. Coverage at least 10×(%), the rate of the whole genome covered by at least 10×sequencing data.
6. Coverage at least 20×(%), the rate of the whole genome covered by at least 20×sequencing data.
