# Supplementary material for: The Antarctic Moss Pohlia nutans Genome Provides Insights Into the Evolution of Bryophytes and the Adaptation to Extreme Terrestrial Habitats
Source: Front Plant Sci. 2022 Jun 17;13:920138. doi: 10.3389/fpls.2022.920138 (PMC9247546; doi:10.3389/fpls.2022.920138)
Supplement: Supplementary Table 1 — Comparison of BUSCO assessment of genome annotation among four bryophytes. [file Data_Sheet_1.zip › Data Sheet 1/Table 3 (25).docx]

**Supplementary Table 3.** Identification of non-coding RNA genes in the *Pohlia nutans* genome.

| Type |  | Copy | Average length(bp) | Total length(bp) | % of genome |
| --- | --- | --- | --- | --- | --- |
| miRNA |  | 55 | 120 | 6590 | 0.000942 |
| tRNA |  | 3740 | 76 | 283153 | 0.040458 |
| rRNA | rRNA | 545 | 185 | 101033 | 0.014436 |
|  | 18S | 28 | 1379 | 38612 | 0.005517 |
|  | 28S | 23 | 186 | 4280 | 0.000612 |
|  | 5.8S | 28 | 148 | 4144 | 0.000592 |
|  | 5S | 466 | 116 | 53997 | 0.007715 |
| snRNA | snRNA | 999 | 139 | 138384 | 0.019773 |
|  | CD-box | 330 | 124 | 40840 | 0.005835 |
|  | HACA-box | 19 | 128 | 2434 | 0.000348 |
|  | splicing | 650 | 146 | 95110 | 0.01359 |
|  | scaRNA | 0 | 0 | 0 | 0 |
