# Supplementary material for: The Antarctic Moss Pohlia nutans Genome Provides Insights Into the Evolution of Bryophytes and the Adaptation to Extreme Terrestrial Habitats
Source: Front Plant Sci. 2022 Jun 17;13:920138. doi: 10.3389/fpls.2022.920138 (PMC9247546; doi:10.3389/fpls.2022.920138)
Supplement: Supplementary Table 1 — Comparison of BUSCO assessment of genome annotation among four bryophytes. [file Data_Sheet_1.zip › Data Sheet 1/Table 4 (17).docx]

**Supplementary Table 4.** Summary of transposable elements (TEs) identified in the *Pohlia nutans* genome.

|  | Repbase TEs | | TE protiens | | De novo | | Combined TEs | |
| --- | --- | --- | --- | --- | --- | --- | --- | --- |
| Type | Length (bp) | % in genome | Length (bp) | % in genome | Length (bp) | % in genome | Length (bp) | % in genome |
| DNA | 9894191 | 1.41 | 8619482 | 1.23 | 158732024 | 22.68 | 165699768 | 23.68 |
| LINE | 1750810 | 0.25 | 3289029 | 0.47 | 41887900 | 5.99 | 43834832 | 6.26 |
| SINE | 51913 | 0.01 | 0 | 0 | 5573433 | 0.8 | 5623543 | 0.8 |
| LTR | 21127156 | 3.02 | 23589949 | 3.37 | 196663517 | 28.1 | 201379172 | 28.77 |
| Satellite | 198274 | 0.03 | 0 | 0 | 4143736 | 0.59 | 4329742 | 0.62 |
| Simple_repeat | 0 | 0 | 0 | 0 | 194055 | 0.03 | 194055 | 0.03 |
| Other | 3695 | 0 | 453 | 0 | 0 | 0 | 4148 | 0 |
| Unknown | 158716 | 0.02 | 26397 | 0 | 123506332 | 17.65 | 123652651 | 17.67 |
| Total | 32137309 | 4.59 | 35513045 | 5.07 | 435620656 | 62.24 | 446472280 | 63.79 |

Note: Repbase TEs, the result of RepeatMasker program based on Repbase database; TE proteins, the result of RepeatProteinMask program based on known TE-related protein of Repbase; *De novo*, the result of RepeatMasker by using library predicted by *De novo* methods; Combined TEs, combine the results of Repbase TEs, TE proteins, and *De novo*. The final non-redundant repeat sequences were obtained by integrated together overlapping TEs from both *de novo* and homology-based predictions.
