# Supplementary figures and images for: The Antarctic Moss Pohlia nutans Genome Provides Insights Into the Evolution of Bryophytes and the Adaptation to Extreme Terrestrial Habitats
Source: Front Plant Sci. 2022 Jun 17;13:920138. doi: 10.3389/fpls.2022.920138 (PMC9247546; doi:10.3389/fpls.2022.920138)

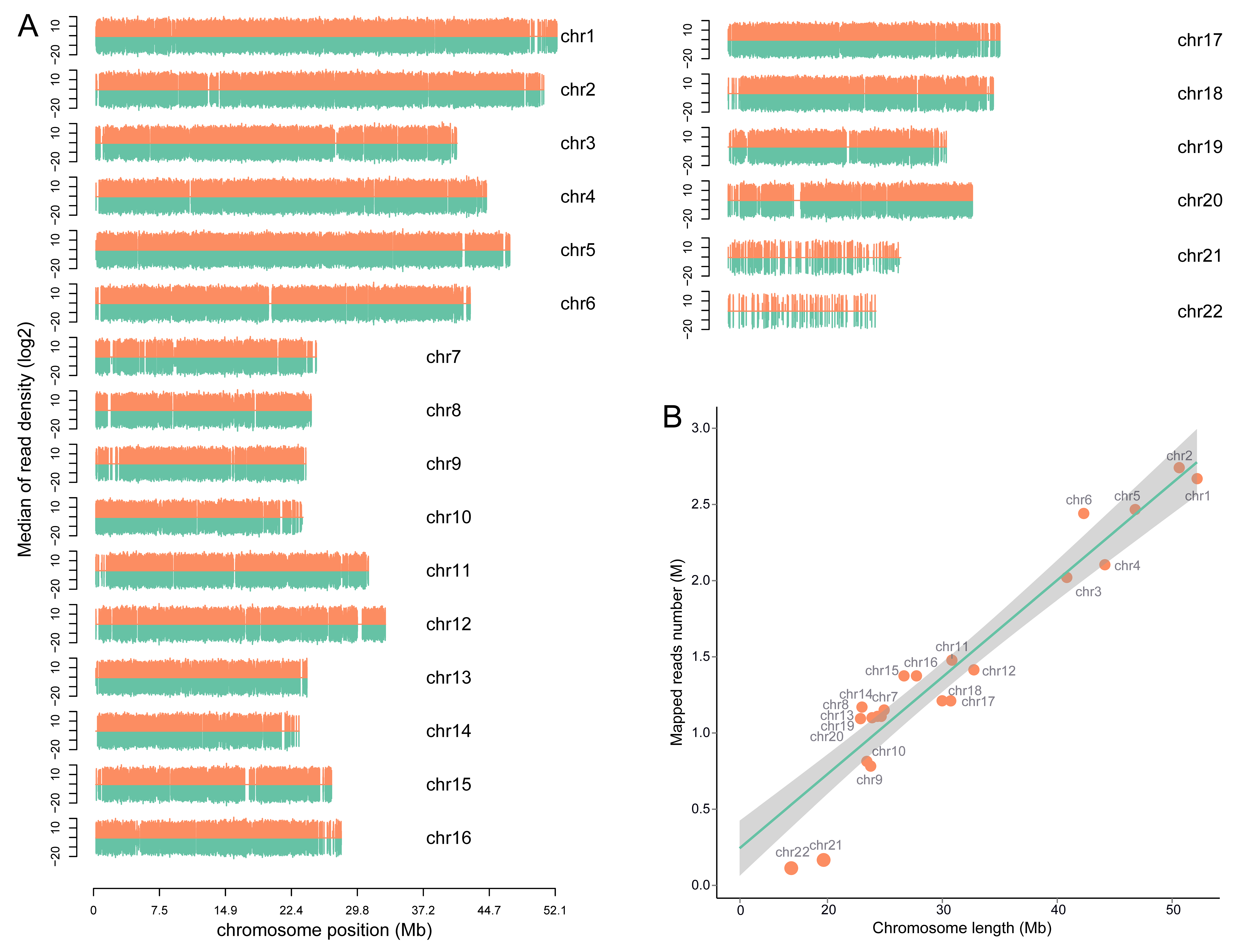

Supplement: Supplementary Figure 1 — Mapping analysis of transcriptome sequencing data. (A) The density of Illumina paired-end reads from transcriptome data in 22 chromosomes of Pohlia nutans. (B) Number of mapped reads from transcriptome sequencing in 22 chromosomes of P. nutans. Transcriptome sequencing data from cold stress were used for this assessment. Fix layout: justify the text. [file Data_Sheet_2.zip › Data Sheet 2/Supplementary Figure 1.TIFF]

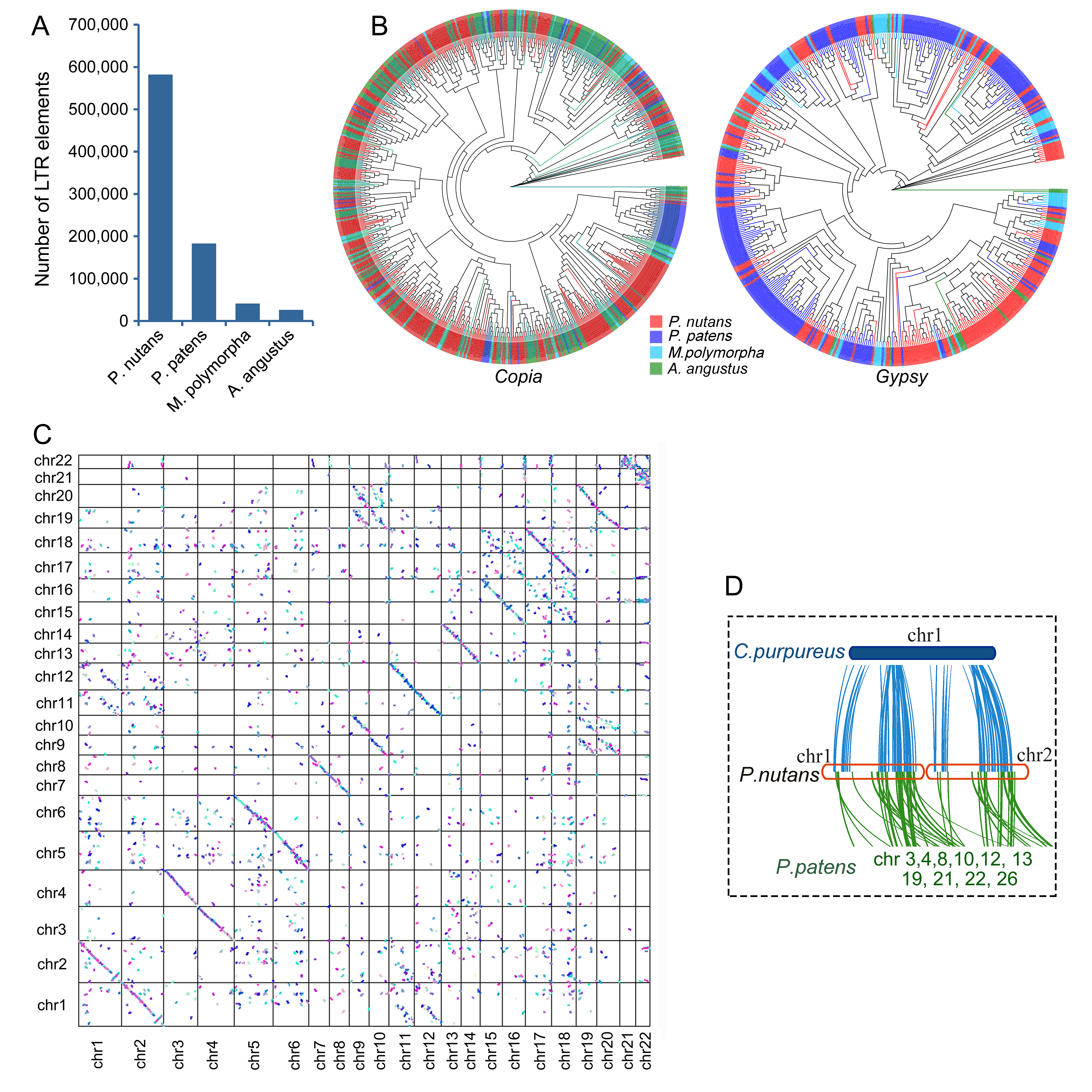

Supplement: Supplementary Figure 1 — Mapping analysis of transcriptome sequencing data. (A) The density of Illumina paired-end reads from transcriptome data in 22 chromosomes of Pohlia nutans. (B) Number of mapped reads from transcriptome sequencing in 22 chromosomes of P. nutans. Transcriptome sequencing data from cold stress were used for this assessment. Fix layout: justify the text. [file Data_Sheet_2.zip › Data Sheet 2/Supplementary Figure 2.TIF]

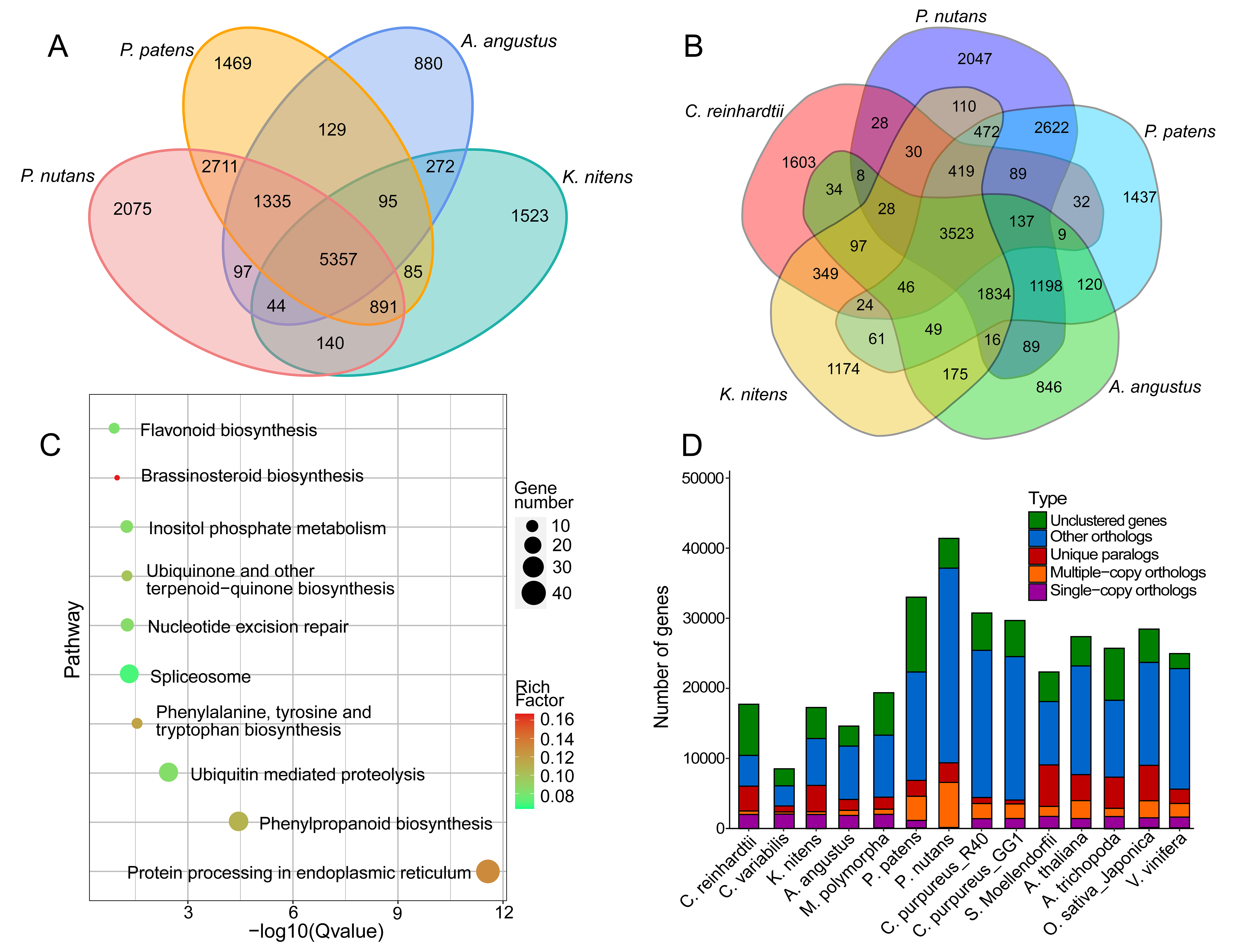

Supplement: Supplementary Figure 1 — Mapping analysis of transcriptome sequencing data. (A) The density of Illumina paired-end reads from transcriptome data in 22 chromosomes of Pohlia nutans. (B) Number of mapped reads from transcriptome sequencing in 22 chromosomes of P. nutans. Transcriptome sequencing data from cold stress were used for this assessment. Fix layout: justify the text. [file Data_Sheet_2.zip › Data Sheet 2/Supplementary Figure 3.TIF]

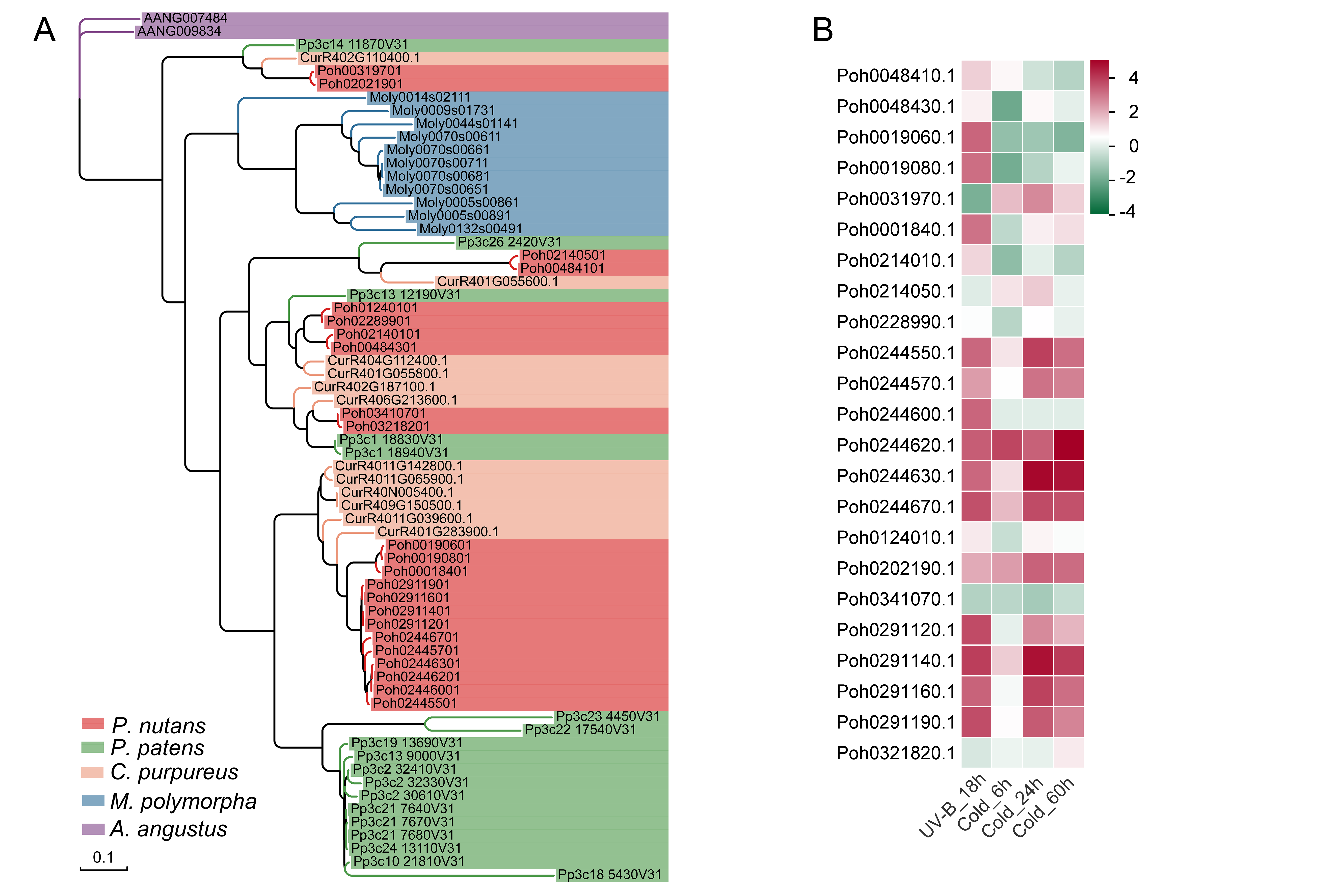

Supplement: Supplementary Figure 1 — Mapping analysis of transcriptome sequencing data. (A) The density of Illumina paired-end reads from transcriptome data in 22 chromosomes of Pohlia nutans. (B) Number of mapped reads from transcriptome sequencing in 22 chromosomes of P. nutans. Transcriptome sequencing data from cold stress were used for this assessment. Fix layout: justify the text. [file Data_Sheet_2.zip › Data Sheet 2/Supplementary Figure 4.TIF]

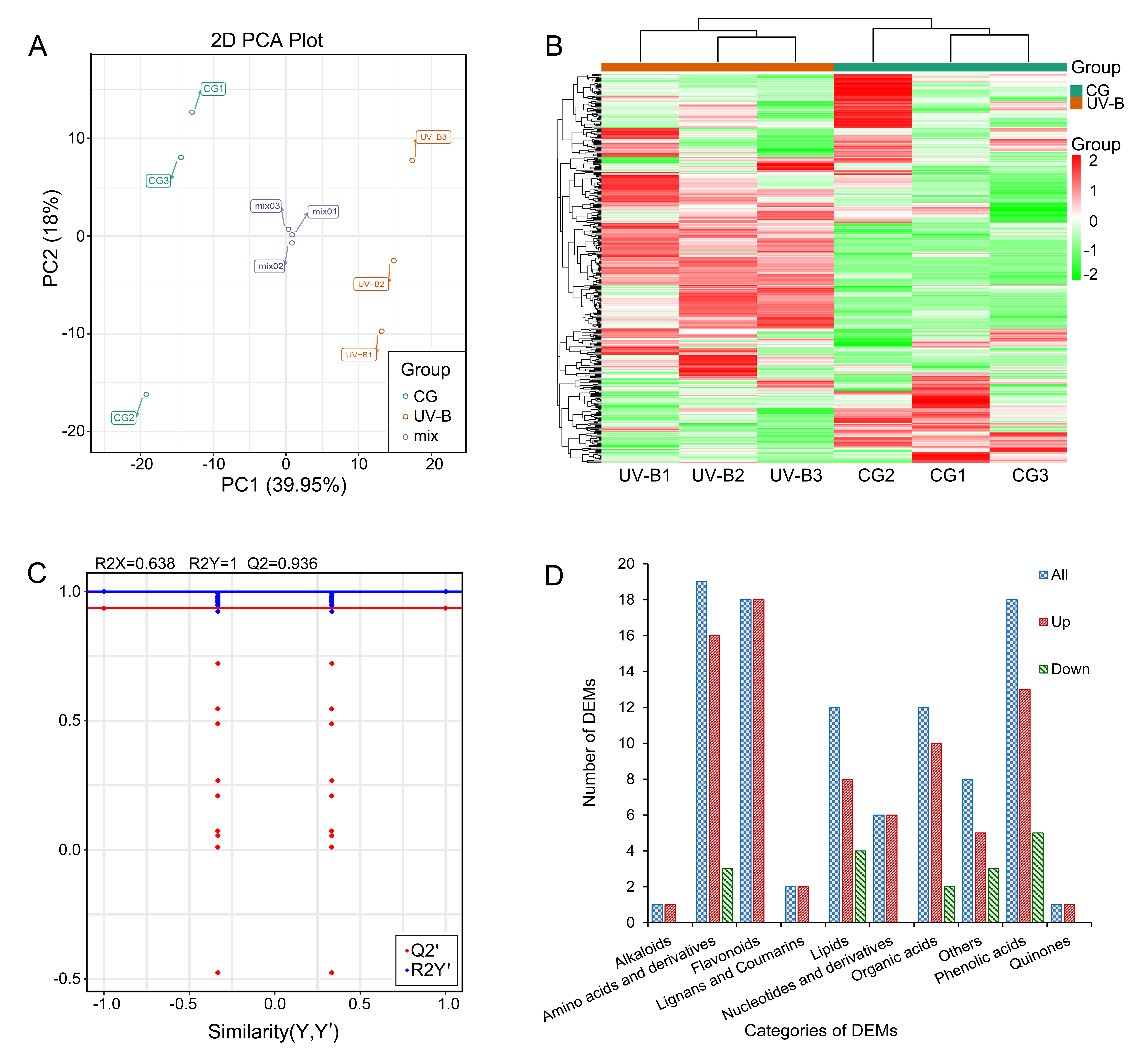

Supplement: Supplementary Figure 1 — Mapping analysis of transcriptome sequencing data. (A) The density of Illumina paired-end reads from transcriptome data in 22 chromosomes of Pohlia nutans. (B) Number of mapped reads from transcriptome sequencing in 22 chromosomes of P. nutans. Transcriptome sequencing data from cold stress were used for this assessment. Fix layout: justify the text. [file Data_Sheet_2.zip › Data Sheet 2/Supplementary Figure 5.TIF]
